# A Recent Progress in the Leachate Pretreatment Methods Coupled with Anaerobic Digestion for Enhanced Biogas Production: Feasibility, Trends, and Techno-Economic Evaluation

**DOI:** 10.3390/ijms24010763

**Published:** 2023-01-01

**Authors:** Muzammil Anjum, Mobeena Anees, Samia Qadeer, Azeem Khalid, Rajeev Kumar, Mohamed. A. Barakat

**Affiliations:** 1 Environmental Sciences, Institute of Soil and Environmental Sciences, PMAS Arid Agriculture University, Rawalpindi 46300, Pakistan; 2 Department of Environmental Sciences, Allama Iqbal Open University, Islamabad 44310, Pakistan; 3 Department of Environmental Sciences, Faculty of Meteorology, Environment and Arid Land Agriculture, King Abdulaziz University, Jeddah 21589, Saudi Arabia

**Keywords:** landfill leachate, pretreatment, hybrid-anaerobic treatment, bioenergy, economic assessment

## Abstract

Landfill leachate (LFL) treatment is a severe challenge due to its highly viscous nature and various complex pollutants. Leachate comprises various toxic pollutants, including inorganic macro/nano components, xenobiotics, dissolved organic matter, heavy metals, and microorganisms responsible for severe environmental pollution. Various treatment procedures are available to achieve better effluent quality levels; however, most of these treatments are nondestructive, so pollutants are merely transported from one phase to another, resulting in secondary contamination. Anaerobic digestion is a promising bioconversion technology for treating leachate while producing renewable, cleaner energy. Because of its high toxicity and low biodegradability, biological approaches necessitate employing other techniques to complement and support the primary process. In this regard, pretreatment technologies have recently attracted researchers’ interest in addressing leachate treatment concerns through anaerobic digestion. This review summarizes various LFL pretreatment methods, such as electrochemical, ultrasonic, alkaline, coagulation, nanofiltration, air stripping, adsorption, and photocatalysis, before the anaerobic digestion of leachate. The pretreatment could assist in converting biogas (carbon dioxide to methane) and residual volatile fatty acids to valuable chemicals and fuels and even straight to power generation. However, the selection of pretreatment is a vital step. The techno-economic analysis also suggested the high economic feasibility of integrated-anaerobic digestion. Therefore, with the incorporation of pretreatment and anaerobic digestion, the process could have high economic viability attributed to bioenergy production and cost savings through sustainable leachate management options.

## 1. Introduction

Leachate is a highly toxic liquid generated regularly in landfills and represents a severe threat to the natural environment and ecosystem [1]. Due to its extreme toxicity, the leachate produced from waste must be collected and treated prior to being discharged into the environment or transmitted to wastewater treatment plants [2,3]. The leachates combine organic and inorganic substances, including nitrogen compounds and heavy metals. The age of the landfill determines its constitution through the kind of trash dumped, climatic conditions, and other factors [4]. Landfills are being overwhelmed with infectious COVID-19 waste due to the recent COVID-19 epidemic, which (with time) may cause space crushing, unauthorized dumping, and the release of toxic pollutants [5,6]. This may also produce additional leachate toxicity and infectious characteristics. A wide range of organic, inorganic, and pathogenic toxins are poorly managed, resulting in lasting damage to land and water [1,5].

Consequently, collecting and recovering LFL is a proactive approach to safeguarding the ecosystem [7]. The dissolved organic matter accounts for 80% of leachate’s total organic carbon content, ranging from 20 g/L or even more [8]. Nevertheless, seven elements influence leachate qualities: the hydrogeology of the location; climatic conditions; landfill age; moisture at the dumping site; trash content; landfill engineering; and landfill operations [8,9]. With each passing day, solid waste management becomes a bigger problem for municipal governments and waste management companies. In Saudi Arabia, the waste generation is up to 1.8 kg per capita per day. Solid waste generation in Saudi Arabia’s three largest cities, Dammam, Jeddah, and Riyadh, exceeds six million tons annually, indicating the scale of local governments’ challenges. More than 75 percent of the population lives in cities, making it imperative for the government to improve the country’s recycling and waste management situation [9]. Thus, mass waste production puts more pressure on landfills and leachate production daily.

Many researchers have investigated different ways to treat leachate successfully to avoid the detrimental effects of produced leachate. The anaerobic method, in particular, is one of the most effective strategies for treating high-strength organic wastes. This bioprocess functions effectively even at high organic loading rates of waste, making significantly lower remaining sludge and more biogas [10]. In high-rate digesters, anaerobic digestion can produce biogas for power generation [11]. In general, anaerobic digestion is one of the prevalent methods for extracting energy from waste that makes organic fertilizer and bioenergy.

The biodegradability of leachate is low due to large molecular weight organics and a high concentration of ammonical nitrogen. Therefore, traditional biological treatment of leachate is challenging [12]. Numerous attempts have been made to comprehend the processes taking place in landfill systems and detect the potentially hazardous chemicals in leachate samples [11,12]. Eggen et al. [13] stated the incidence of emerging contaminants, such as chlorinated alkyl phosphates, N-butyl benzene sulfonamide, diethyltoluamide, personal care products, an anti-inflammatory drug, and polycyclic musk compounds in the water and solid (particle). It has been noticed that biological treatment techniques can eliminate COD and NH^4+^-N, but most trace pollutants remain in the leachate [14]. Poly and perpolyfluoroalkyl substances, bisphenols, organophosphate esters, and other medicines make up the majority of trace organics. Even in contemporary operating (active) or recently-closed landfills, landfill managers are not obliged to screen for the existence of these contaminants [15]. The lack of awareness on the trace organics determination, principally those with endocrine disruptive action, is a substantial awareness gap that must be fulfilled. Additionally, traditional treatment methods are insufficient to eliminate the new pollutants [16], trace organics, and heavy metals. Furthermore, present treatment systems have significant limitations, including the inadequate removal of contaminants, high energy demand, and the development of noxious sludge [17]. In such instances, techniques are urgently needed to improve treatment efficiency.

**Figure 1:**
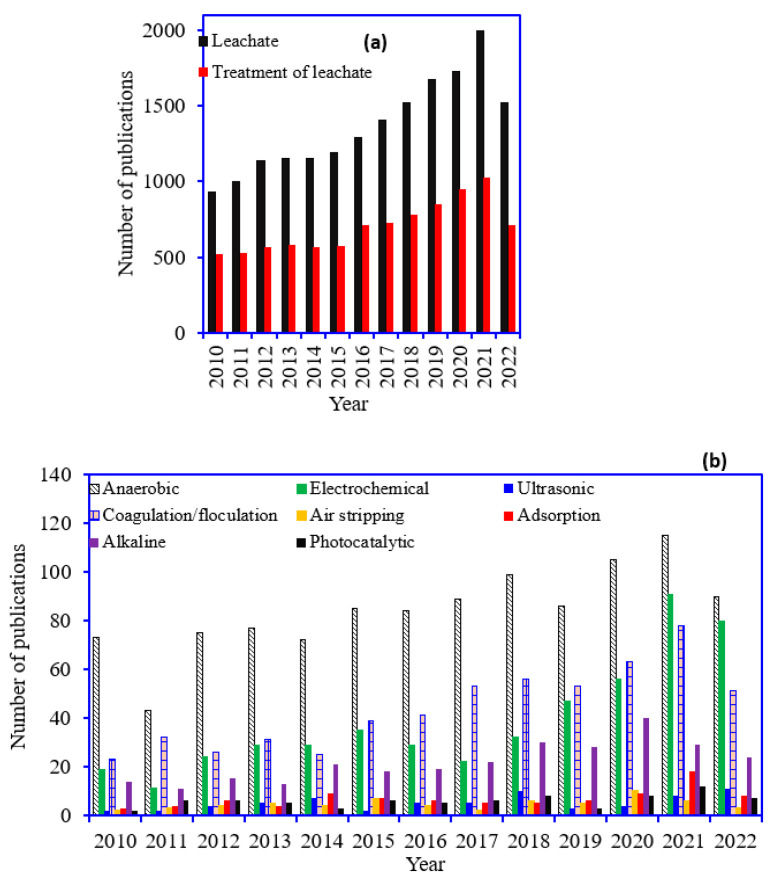
(**a**) Scopus database showing the number of the articles published searched using the keywords “leachate” and “treatment of leachate” and (**b**) number of the articles published searched using the keyword” treatment of leachate using various methods” between the years 2010 to September 2022.

Due to the large polluting loads, the integration of biological, chemical, and physical methods is commonly used to remediate leachates, because a single treatment method does not deliver the complete removal of pollutants. Numerous physical/chemical strategies for treating leachate have been developed, including coagulation-flocculation, membrane filtration procedures, advanced oxidation methods, and so on [18]. Figure 1 shows the number of publications per year using the various keywords in Scopus between the years 2010 to September 2022. Figure 1a demonstrates the number of publications searched using the keywords “leachate” and “treatment of leachate”. The keyword leachate shows the articles published in various fields, such as the management, analysis, and treatment of leachate. While the keyword treatment of leachate showed the articles related to the multiple methods used for treating or purifying leachate wastewater. The articles published on the treatment of leachate using various processes are mentioned in Figure 1b. The literature review revealed that anaerobic treatment is the most applied method for leachate purification. Electrochemical, coagulation/flocculation, and alkali treatments were also used for leachate decontamination. The unique features of the leachates mainly determine the optimal treatment procedures under consideration [19]. Advanced Oxidation Processes (AOPs) could be a feasible alternative for abolishing recalcitrant toxic compounds, using the action of strong oxidizing species like hydroxyl (HO^•^) radicals [12]. For instance, the use of nanomaterials (MnO, Fe, MgO, ZnO, CNT), nano photocatalysts (TiO_2_, ZnO, CdS, ZnS:Cu, CdS: CdS:Eu, Mn), and electrocatalysts (Pt, Pd), coupled with biological processes, is also an option. Alternative leachate management approaches have also been proposed, such as recirculating generated leachate through discarded trash. The main advantage of this strategy is its ease of use and minimal capital and operating expenses. Because microorganisms inhabit most landfills, recirculated leachates are suitable for biological treatment [19]. This review mainly focuses on the feasibility of combining two processes, i.e., pretreatment and anaerobic digestion for leachate treatment, to achieve the benefits.

## 2. Leachate Toxicity and Environmental Impacts

**Figure 2:**
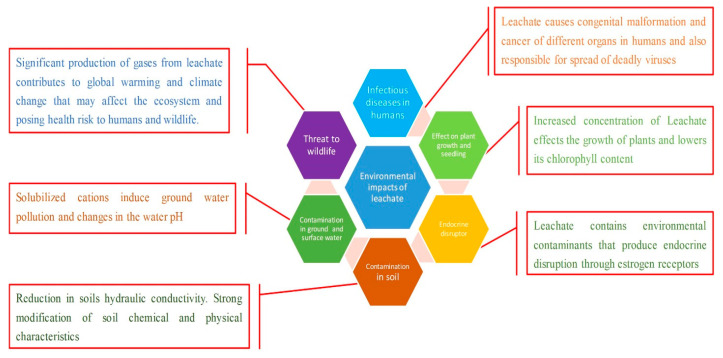
Impacts of leachate on human health and the environment.

Many studies have been conducted to assess the risks of surface and subsurface water contamination and the corresponding influence on LFL health. Over 200 organic compounds have been discovered in LFL, with 35 chemicals potentially harmful to the environment and human health. The leachate characteristic is summarized in Table 1. The aqueous solution known as LFL contains four different types of contaminants. The natural dissolved organic matter, such as refractory chemicals, heavy metals (Hg, Cu, Pb, Ni), cations and anions, such as SO_4_^2−^, Cl^−^, Fe^3+^, Al^3+^ etc., and xenobiotic compounds (PCBs), dioxins, halogenated organics, and so forth, are all present in leachate [20]. Figure 2 summarizes various leachate contaminants and their impacts on the environment and human health [19,20,21].

The illegal dumping of COVID-19 infectious waste in landfill, especially in developing countries, is also posing a risk of leachate due to the toxicity of infectious plastic waste (PPEs). These abandoned items produced a large amount of plastic waste, which ended up in nature as microplastics and nano plastics, both harmful to the ecosystem. These micro- and nano-plastics also serve as vectors for pathogenic pollutants [22]. Some recent studies have shown the decomposition of infectious coronaviruses might be accelerated by wastewater [23]. However, in practical implementations, sewers are more complex systems than bulk wastewater, making it more challenging to comprehend the fate of the coronavirus. The existence of trace organics in landfill leachate is apparent, raising worries about their escape into the ecosystem. The occurrence of trace organics in landfill leachate significantly increases the risk to the environment and public health. Although trace organics such as endocrine-disrupting chemicals have been discovered in landfill leachate, little is known about their effect on the environment and fate in the environment around the dump [24]. This lack of understanding represents a substantial knowledge gap that requires additional investigation. A recent study has also revealed traces of organics found in landfill leachate, including phthalates, pharmaceuticals, and personal care products [25].

ARGs are also of particular concern as emerging pollutants because of their hazards to human health and environmental sustainability [26,27]. Despite several reports on antibiotic-resistant bacteria discharged from treated municipal wastewater, data on ARBs and ARGs from MSW landfill leachate is rarely published. A few studies have suggested that leachate is a major hotspot for ARG accumulation and propagation [28,29]. Landfill is the most frequent method of MSW disposal worldwide, and food waste leachate is regarded as an important ARGs reservoir [28]. The issue has received insufficient attention, and therefore it is essential to consider the toxicity and treatment of ARGs in landfill leachate [27].

**Table 1:** Characteristics of different types of leachate [8,28,29,30].

Parameter	Unit	Type of Landfill Leachate		
		Young (0–5 y)	Intermediate (5–10 y)	Mature (10–20 y)
COD	mg/L	>10,000	4000–10,000	<4000
Period		Transition	Methane fermentation	Final maturation
BOD	mg/L	0.5–1.0	0.1–0.5	<0.1
pH		<6.5	6.5–7.5	>7.5
Organic compounds	%	80 VFA	5–30 VFA+HFA	High fulvic fraction
NH_3_-N	mg/L	<400	NA	>400
Heavy Metals	mg/L	Low to medium	Low	Low
Biodegradability		Important	Medium	Low
Stability		Unstable	Moderately stable	stable
Phase		acidogenic	methanogenic	Stabilization/methanogenic
Color appearance		Light yellow	Light yellow	Dark black green

Municipal solid waste (MSW) leachate discharge has the potential to severely damage groundwater and surface water. Despite an excellent landfill site selection and monitoring network architecture, the effects of waste leachate on the environment, particularly on groundwater quality, have been repeatedly recognized by several studies. Even in the most excellent liner, the leachate intrusion into groundwater is a severe problem because of normal degradation to the leachate collecting systems [21,31]. Furthermore, even when the leachate is not substantially polluting, the pH of groundwater may alter as a result of the leachate intrusion [32], resulting in metal dissolution from the subsurface matrix [33] into the groundwater. Therefore, groundwater quality monitoring systems are the key indicators for measuring the possibility and sternness of pollution problems, and they are crucial to the entire design of a landfill.

Organic matter, pH shifts, and the redox conditions of the aquatic medium of the soil can extract various metals through the solubilization of multiple minerals [34]. As a result, risk evaluations and environmental legislation for contaminated soils are based on batch metal extractions experiments, presuming that the findings are linked to the risk of metal leaching into groundwater or plant uptake. Adding pollutants from leachate into the soil can seriously damage plant growth. For instance, MSW leachate pollutants can stifle wheat seedlings’ growth. The contaminants found in wheat seeds could significantly impact plant growth, having reduced growth due to the application of MSW leachate. These physiological reactions might differ depending on the amount of MSW leachate present [31].

## 3. Anaerobic Digestion: Viability for Leachate Treatment

Anaerobic digestion is a biochemical process that occurs under anoxic conditions. It involves the coordinated action of several different types of bacteria that break down organic molecules into a gaseous mixture, mainly containing CH_4_ and CO_2_. It is a popular method for lowering the amount of biomass, while treating numerous types of organic substrates such as sewage sludge, municipal solid waste, manure, and lignocellulosic biomass, with additional methane extraction as an economical energy source [35]. Anaerobic digestion also provides the promising elimination of ARGs in leachate [27]. The methane thus produced as a by-product can be used directly to generate heat, electricity, and other energy purposes. The digestate can be utilized as a fertilizer. Moreover, AD provides significant environmental advantages, including pathogen control, the avoidance of biomass acidification and putrefaction, decreasing pollution, and greenhouse gas control through the stability and control of biomass waste [36].

Various studies reported the anaerobic digestibility of leachate and methane yield under different operating conditions, such as OLR, HRT, temperature, etc., (Table 2). In recent years, the applicability of the digestion system treatment of different types of organic wastes has been examined [18]. Researchers have focused on treating leachate using anaerobic reactors. Furthermore, previous studies have focused on using complicated reactors, including baffled reactors, migrating blanket reactors, granular sludge bed bioreactors, hybrid anaerobic reactors, etc., [37,38,39]. Indeed, COD removal rates ranging from 71 to 99% were observed when anaerobic systems were used, with migrating blanket reactor reactors having recorded the most significant removal rates [40]. Mokhtarani [38] reported that the effectiveness of COD degradation depended on the organic load rate at a threshold of 6.0 kg of COD/m^3^/d, but drastically reduced above that barrier. These outcomes, however, contradict those reported by Liu et al. [37] that an OLR of about 22 kg COD/m^3^/d was the best. Numerous factors can affect the anaerobic digestion of leachate; however, pH, recirculation, and microbial diversity play the most crucial role.

Unlike municipal wastewater, leachate treatment is more complicated and complex. Traditional bio-treatment plants, for instance, sequencing batch reactor systems, were designed to remove nitrogen, phosphorus, and organic carbon, but exhibited low removal efficiency for antibiotics and ARGs [59]. Moreover, the organic waste under anaerobic conditions in landfill produces refractory chemicals such as fulvic acids and humic acid, which directly limits the bio-degradability of leachate [60]. This may be due to the scarcity of easily accessible substrate and the presence of NH_3,_ reportedly up to 3000 mg/L, attributed to the lower action of the microorganisms [57]. Therefore, biological treatment is usually not advised for leachate treatment, and a modification of the process may be required to accomplish the anaerobic digestion of leachate.

### Anaerobic Co-Digestion of Leachate

The anaerobic digestion process appears ideal for waste treatment because of its high carbohydrate, protein, and fat content. However, various constraints remain, such as forming volatile fatty acids and a trace metal shortage [61]. These limitations make it difficult for a single substrate through the anaerobic process to accomplish stable organics for methane conversion at large loading rates [53]. As an alternative option for enhancing digestion efficiency, co-digestion adds up more than one organic waste stream as substrates simultaneously, appearing potent. Co-digestion can increase process performance by improving nutritional balance and the synergetic effect between organic substrates [46]. High ammonia concentrations in mature leachate can improve the system’s ability to buffer pH by reducing acidification risk [62,63]. Furthermore, during the co-digestion process, mature leachate can provide nutrients for microbial development and activity [53,64].

Landfill leachate has frequently been utilized as a co-digestion substrate for septage, sewage sludge, household wastewater treatment, and food waste, among other things [46,65]. In a study by Liu et al. [53], the anaerobic digestion performance in an expanded granular sludge blanket reactor was enhanced by the co-digestion of mature leachate and food waste. The methane production rate of 5.87 L/L/d was obtained with a COD removal rate of roughly 80% in the reactor. The addition of mature leachate supplied ammonium to minimize acidification and trace metals for microbial growth, where all the phases of anaerobic digestion were improved. Where in another study by Takeda et al. [66] on the anaerobic co-digestion of various landfill leachate and crude glycerol, the organic matter removal efficiency of up to 90.15% and the specific biogas generation of 403.15 mL/g VSS in 33.2 days at a glycerol concentration of 1.71% and a substrate/inoculum ratio of 0.37 g COD/g VSS, were obtained. Another recent study developed a laboratory-scale up-flow anaerobic sludge blanket reactor for the co-digestion of landfill leachate and acid mine drainage. It was found that the maximum removal efficiency for COD and sulfate was 83% and 78%, respectively, at hydraulic retention time of 20 h.

## 4. Hybrid Treatment

The leachate produced in landfill has a complex composition. The leachate content principally depends upon the organic waste type, the treatment method, the level of maturity, and the amount of oxygen saturation in the system [16,45]. Leachates exhibit significant fluctuations due to their chemical composition and volumetric flow. Various factors, such as the landfill’s age, the type of waste dumped, and the waste quality and hydrogeological conditions, all impact the content and concentration of contaminants. As landfill ages, there is a transition from an initially shorter duration of aerobic degradation to a more extended process period [19]. Studies showed that leachate with a low maturation degree has two main characteristics: high COD values up to or higher than 50,000 g/L; and high COD to BOD ratios. Volatile fatty acids are produced when volatile solids are hydrolyzed and acidified, making up the most biodegradable organic matter. Due to the high VFA content, the pH of such leachates is often acidic. On the other hand, mature leachates contain low biodegradable organic fractions (BOD_5_/COD: 0.05–0.2), alkaline pH, lower COD concentration, and greater quantities of resistant chemicals [67].

Older leachates typically contain bio-refractory pollutants that are resistant to normal biological processes. However, leachates’ high ammonia content may inhibit microorganism growth [19]. Before anaerobic digestion, the leachate is pretreated to accelerate the hydrolysis reaction and complete the breakdown of the harmful organic compounds. This results from microbial cells utilizing smaller organic molecules as substrates more quickly and efficiently [68]. The large organic molecules in the leachate can be hydrolyzed or broken down by the pretreatment processes, which can use mechanical, biological, chemical, and physical methods or a mix of these techniques [69]. Mechanical shear-induced disintegration can be conducted by high-speed agitation or sonication, photocatalysis, high-temperature treatment, air stripping, freeze-thawing, electrocatalysis, microwave irradiation, ozonation, acids or alkalis, biological hydrolysis, and ultrasound irradiation [16,53,54]. The leachate contains numerous types of pollutants that can cause toxicity to the environment. The treatment of leachate has a varying potential of removing contaminants, summarized in Table 3.

## 5. Pretreatment-Anaerobicdigestion(AD)Ofleachate

### 5.1. Electrochemicalpretreatment-AD

Electrochemical treatment is a popular technology application for treating leachate [78]. Various chemicals such as antibiotics, nitrite-N, ammonium-N, and others, may be efficiently removed using this method [54,56,57]. Electrochemical oxidation provides the advantages of the absence of additional chemicals and the lack of need to dispose of secondary waste products. Moreover, it offers the breakdown of large molecular components into smaller ones, complete mineralization, easy operation, and simple instruments. Thus, it could be a potential approach for enhancing the biodegradability of leachate [42]. Electrochemical oxidation has been considered as pretreatment to subsequently anaerobic digestion process to improve methane production.

Electrochemical oxidation is documented as the electrolysis and oxidation-reduction of organic pollutants in liquid media through transferring the electrons between electrolytic solution and electrodes, under the impact of an external electric field between the cathode and anode [55,79]. The existence of hydroxyl radicals and various oxidizing species such as HClO^∙^, H_2_O_2_, and Cl_2_ on the anode and electrolyte, assist in removing biologically recalcitrant molecules such as aromatic compounds [41]. Pollutants can directly oxidize the anode plate by transferring electrons to its surface during electrochemical oxidation, or oxidants can be produced when electrolyte ions are anodized, such as available chlorine, persulfate, perphosphate, hydroxyl radicals, and percarbonate [35,54]. Electrochemical treatment can remove several pollutants from leachate in a single run. For instance, Song et al. [80] found that electrochemical oxidation under optimal conditions removed total organic carbon, chromaticity, and total nitrogen, by more than 70%, whereas ammonia nitrogen was removed by up to 99%. Urtiaga et al. [81] investigated that leachate samples were electro-oxidized to remove 17 poly- and perfluoroalkyl compounds present in the leachate. It was found that electro-oxidation operating at 800 A/m^⁷^ for 6 h effectively lowered the concentration of 17 poly- and perfluoroalkyls by 95%.

To achieve substantial effectiveness, the electrodes in the electrolytic system must have a large electrochemical range to maintain stability over a long period. As a result, the electrode material is vital to the electrochemical process. For instance, because of their high oxygen transformation over potential, lead boron-doped-diamond electrodes, and tin oxides promote total mineral deposits of organic molecules. In contrast, platinum, iridium, titanium oxide, and ruthenium anodes exhibit high electrocatalytic activity, resulting in the rapid disintegration of organic molecules at lower potentials [80]. In an aqueous solution, the electrochemical process employs two major mechanisms [42]: (1) electrochemical conversion, which includes partly oxidizing refractory organic molecules to create more degradable chemicals that are ideal substrates for biological therapies; and (2) electrochemical combustion converts organic molecules entirely into water, CO_2_, and other inorganic substances. Equations (1) to (7) show the indirect oxidation during electro-oxidation. The organic molecules can be oxidized by OCl^-^ in aqueous media. The mechanism of oxidation of leachate compounds is given as follows:

At anode

2Cl^−^ → 2e^−^ + Cl_2_ (1)

6H_2_O + 12HOCl → 4ClO_3_^−^ + 3O_2_ + 8Cl^−^ + 24H^+^ + 12e^−^ (2)

2H_2_O → O_2_ + 4e^−^ + 4H^+^ (3)

Bulk reactions

Cl_2_ + H_2_O → H^+^ + Cl^−^ + HOCl (4)

HOCl → OCL^−^ + H^+^ (5)

At Cathode

2H_2_O + 2e^−^ → 2OH + H_2_ (6)

2H_2_O + 2e^−^ + OCl^−^ (7)

**Figure 3:**
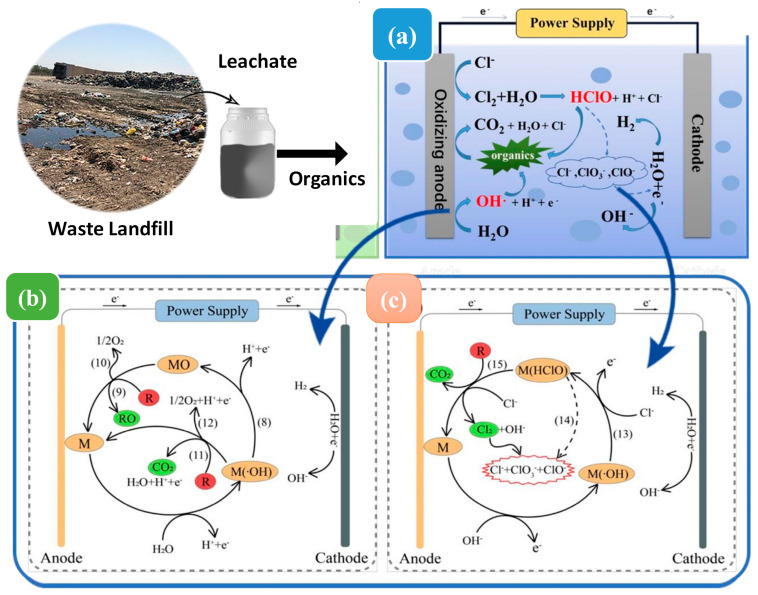
Electrochemical oxidation mechanism. (**a**) hydroxyl radical-mediated indirect oxidation (**b**), active chlorine-mediated indirect oxidation (**c**) (modified and acquired from [72].

A schematic explanation of the electrochemical reaction is illustrated in Figure 3. Electrochemical oxidation of leachate removes pollutants primarily by: (1) direct oxidation, i.e., direct electron transfer to the oxidizing anode surface; and (2) indirect oxidation by producing electroactive compounds. Various studies have been performed using electrochemical methods with a biological/anaerobic digestion process for landfill leachate treatment. For instance, the combination of biological and electrochemical treatment leachate examined by [82], based on an improved electrochemical oxidation mechanism, suggested a two-step electrochemical method. An electrochemical pulse method was used in the first step. In the second condition, 12.5 mA/cm^2^ of current density was applied, and the pollutants were further eliminated by electrolytic deposition and oxidation. In another study, Pasalari et al. [42] showed that during electrochemical pretreatment, when the current density was raised from 10 mA/cm^2^ to 40 mA/cm^2^, sCOD increased from 320 mg/L to 1165 mg/L, and total volatile fatty acid from 172 mg/L to 742 mg/L. In a later test, the anaerobic digestion reactor fed with leachate produced the maximum methane output of 0.292 L/g sCOD removal.

### 5.2. Ultrasonic Pretreatment-AD

Ultrasound can be used for leachate pretreatment before anaerobic procedures to improve anaerobic reactor performance by providing more bioavailable organic substrate and aiding the hydrolysis stage. Increasing the soluble organic matter percentage and lowering particle size, the pretreatment procedure assists in the disintegration of solid materials [83]. Ultrasonic waste pretreatment has improved anaerobic processes and methane production rates [84].

**Figure 4:**
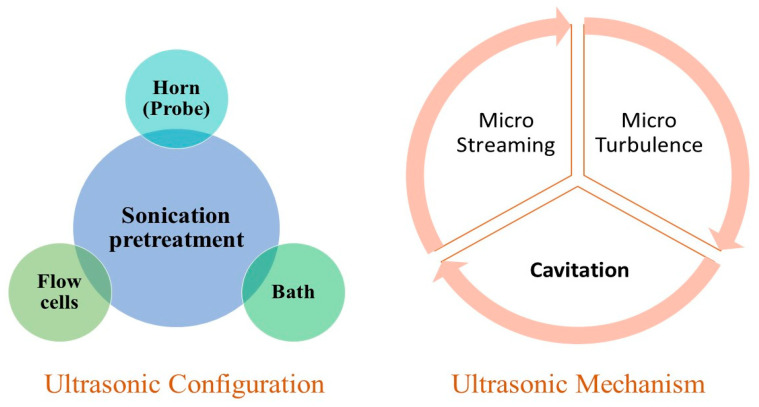
Ultrasonic pretreatment configuration and mechanism requirements.

Sonication is yet another technique for injecting energy into a reaction system to achieve the required biological, chemical, or physical change. Sonication has several possible advantages in leachate pretreatment to facilitate anaerobic digestion, such as the degradation of large organic molecules into short organics that microbial cells can easily uptake, thus resulting in enhanced biogas generation. Furthermore, the hydrolysis of the leachate produces haloacetic acid and trihalomethanes, both of which could be used as disinfectants [85]. Ultrasonication’s main advantages are simple, quicker degradation kinetics, better ambient conditions for bulk reaction mixture, lower hazardous by-product formation, and strong compatibility and synergism with traditional treatment procedures [86]. The mechanism related to the solubilization using low ultrasound frequency is based on acoustic cavitation, which creates hydromechanical shear stress [87]. Because of the cavitation effects, low-frequency ultrasound may also be applied to enhance the rate of hydrolysis of complicated liquid wastes such as leachate. The purpose of ultrasonic pretreatment is to accomplish partial pollutant degradation and to improve biological treatment units. Implementing a low-frequency ultrasound procedure before anaerobic processes could potentially increase liquid waste’s anaerobic treatability by boosting organic matter solubilization and increasing methane production [84]. The cavitation phenomenon is linked to the increase in the sonication process. Cavitation is described as the nucleation, growth, volume oscillations, and final transient collapse of gas or vapor bubbles in a liquid medium induced by pressure changes through ultrasonic wave transmission [88]. Transient cavitation concentrates considerable energy on tiny temporal and spatial dimensions [85]. The bubble implosion creates zones of extreme settings, with heating temperatures exceeding 5000 K and reaching 100 MPa of pressure, resulting in various physicochemical phenomena. Acoustic cavitation is a phenomenon that occurs at a low-frequency range between 20–40 kHz and generates significant shear forces by jet streams acting on the compounds in the medium throughout the cavitation bubble implosion [84]. Figure 4 shows an ultrasonic pretreatment configuration and mechanism requirements.

Various studies have been performed using ultrasonication as a leachate pretreatment before the anaerobic digestion process. Integrating experimental data with cavitation bubble dynamics simulations in the study of Nazimudheen et al. [85] aimed to acquire mechanistic insight into ultrasonic-aided LFL pretreatment (before anaerobic digestion). Sonication created strong micro convection in the system, which accelerated biosolid breakdown and solubilization. However, with high input power, organic matter breakdown occurred in the solution due to oxidizing radicals created by cavitation bubbles, thus resulting in minimal biogas generation in anaerobic digestion. Sonication with low to moderate power input produced solubilization, and disintegration but not complete deterioration, resulting in greater sCOD values for higher biogas production. Neczaj and Kacprzak [89] studied the leachate’s sonification in static conditions using the disintegrator UD-20 at a 22 k field frequency of 12 m for better results sonification of leachate improves COD and ammonia reduction as compared to the control trial. Oz and Yarimtepe [84] used the ultrasonic frequency of 20 kHz to evaluate the solubilization of organic materials in leachate. After 45 min of sonication at 600 W/l, COD/tCOD ratio improved to 63% from 47% (raw leachate). When anaerobic reactors were fed with ultrasonically processed leachate substrate, they produced 40% more biogas than the control reactor.

### 5.3. Coagulation/Flocculationpreatreatment-AD

The leachate coagulation-flocculation technique has been mainly studied utilizing stabilized or biologically pretreated landfill leachates [90]. The CFs have been widely used as a pretreatment to eliminate leachate factors such as color, suspended particles, and high concentrations of organic contaminants before biological treatments. However, little information on the efficacy of these physicochemical methods is available. This method is used to degrade pollutants from leachates that have been somewhat stabilized by recirculation or form newly created leachates. When applied before biological and anaerobic treatment, this method is critical for increasing later leachate biodegradability. Several findings on the examination of coagulation-flocculation for treating LFLs are reported, with the goal of performance optimization, i.e., the choice of the utmost appropriate coagulant, the determination of pH effect and experimental conditions, and the study of flocculent addition. As coagulants, FeSO₄, Al₂(SO₄)₃, FeCl₃, and ferric chloro-sulfate were extensively utilized. The joint action of lime and alum to treat stabilized leachates showed substantial COD removal capabilities [19].

Smaoui et al. [91] reported that the process of coagulation-flocculation increased effluent biodegradability, while reducing COD by 46%. The potential methane assay was used to assess the effect of pretreatments. Coagulation-flocculation produced more biogas than other pretreatments, with a yield of 370 mL/g COD. Some researchers employed ferric chloride, aluminum polychloride, aluminum sulfate, and polyacrylamide polyelectrolytes as coagulants and flocculants for leachate treatment. Using ferric chloride at a dose of 0.6 g Fe L^−1^, up to 73% COD reduction was achieved. Similarly, nonbiodegradable substances were reduced up to 73% while treating aged leachate where the BOD5 equals 670 mg/L and COD: 4800 mg/L) [92]. As a result, reducing nonbiodegradable chemicals in leachate by coagulation and flocculation pretreatment may improve the anaerobic digestion process by introducing more biologically degradable compounds.

### 5.4. Airstrippingpretreatment-AD

Ammonia stripping has been utilized effectively in landfill leachate pretreatment by Lei et al. [77]. Ammonia or air stripping is the extensively used method for removing NH_3_-N from landfill leachate [8]. Following stripping, the concentrated gaseous ammonia can be recovered and absorbed by acidic solid solutions like H_2_SO_4_, producing inorganic compost for farming use [93]. There has been little investigation into the application of ammonia stripping to anaerobically digested wastewater. Smaoui et al. [94] used the biochemical methane potential test to examine the influence of air stripping pretreatments. Stripping provided a higher biogas output than previous pretreatments, reaching 588 mL/g COD. Lei et al. [88] improved ammonia stripping for pretreating anaerobic digestion wastewater from a facility. It was also studied to see if CO_2_ stripping and biogas injection might be used to regulate the pH of the effluent before and after the ammonia stripping process. The optimum Ca(OH)_2_ dose is essential to study the diverse C, N, and P concentrations of digestate. Smaoui et al. [94] stated that the ammonia stripping pretreatment of leachate might achieve an ammonia reduction of 80% and an improvement of C: N to 25, which is an acceptable ratio for anaerobic digestion. This later anaerobic digestion was carried out in a fixed bed reactor with a continuous loading rate of 3.2 g and 2 COD/L/d diluted and raw leachate. The anaerobic digestion process resulted in considerable COD reduction and biogas generation, particularly for the diluted leachate. The raw leachate had a COD removal rate of 78% and a biogas generation rate of 4 L/d, with a methane concentration of 70%. It is also reported in a study on landfill leachate pretreatment by air stripping that the pretreated leachate produced 7 L/day biogas with 75% CH_4_ content. The authors reported that pretreated leachate produced 60% higher CH_4_ than raw leachate. Air stripping decreases the 80% ammonia and a rise in the C to N ratio by 25, which is appropriate for AD. Ammonia reduction help in the growth of microbes with produces more CH_4_.

**Figure 5:**
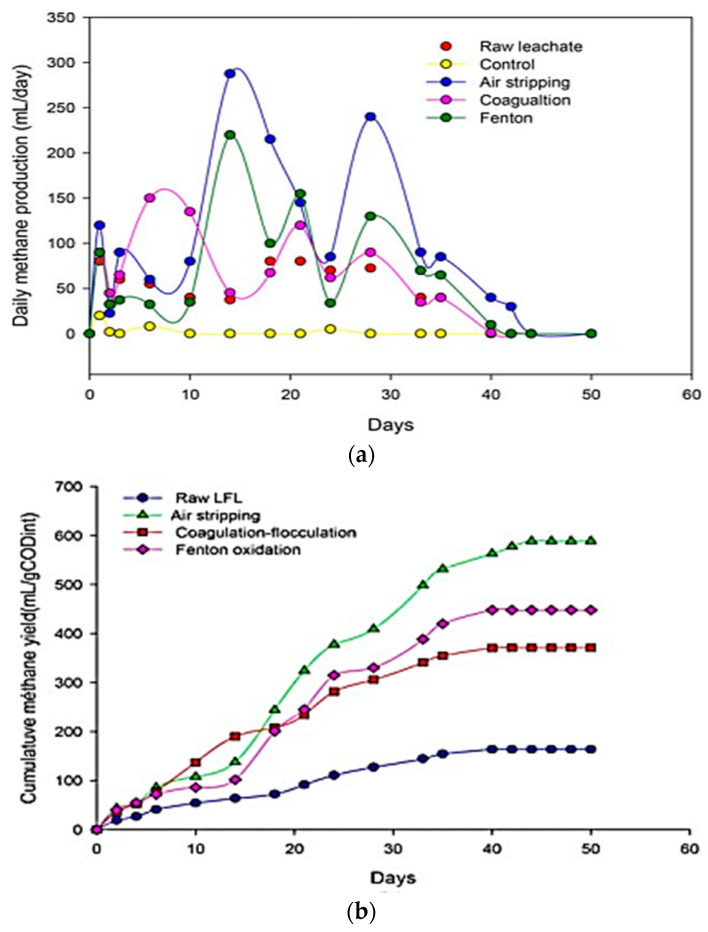
Methane production during batch anaerobic treatment of landfill leachate (**a**) daily methane production rate, (**b**) cumulative methane production [91].

An earlier study by Smaoui et al. [91] compared the effectiveness of coagulation-flocculation, air stripping, and Fenton’s oxidation in the pretreatment of leachate and biogas production. The air-stripping pretreated LFL shows a better output of the CH_4,_ as shown in Figure 5. Although the organic matter removal effectiveness of the coagulation-flocculation and Fenton’s oxidation is much higher than air stripping, compared to air stripping, the CH_4_ production rate is low due to low ammonia reduction by coagulation-flocculation and Fenton’s oxidation. Moreover, a combined pretreatment of LFL by air stripping coupled with agitation and coagulation-flocculation process is more effective, and the elimination of COD, NH_3_-N, and BOD_5_ were up to 71.5%, 96%, and 56.5%, respectively. The BOD/COD ratio was also enhanced from 0.20 to 0.31 [95].

### 5.5. Adsorption Filteration–AD

Adsorption from leachate desorption is frequently used in leachate treatment because it improves the elimination of recalcitrant and toxic organic pollutants [96]. Although it could not directly help improve the solubilization of organic compounds, removing such compounds could hinder the anaerobic digestion of leachate. Mainly, adsorption is used to extract organics and metals from leachate. Granular activated carbon has been widely employed due to increased adsorption capacity, surface area, and improved thermal stability [97]. Pollutants can adhere to the adsorbent’s surface in various ways during adsorption. The adsorbent’s surface has certain features that allow the ascorbate to attach. Under specific conditions, a reversible phenomenon known as desorption happens. Adsorbates can be liberated from the adsorbent’s surface and returned to the liquid during desorption [98].

**Figure 6:**
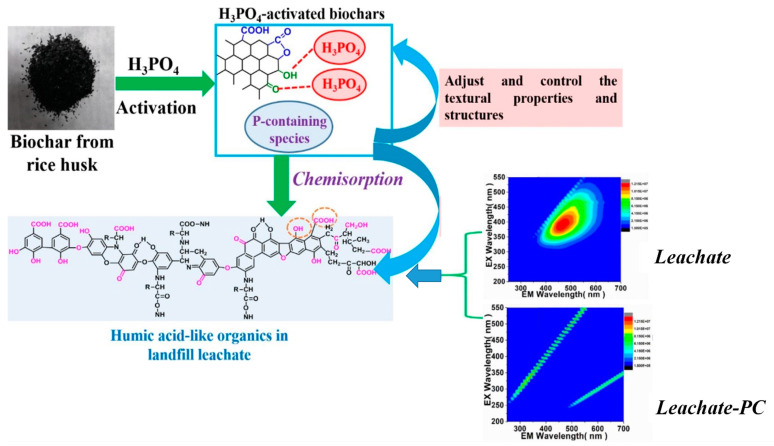
Treatment mechanism through adsorption from landfill leachate by activated biochar [101].

Cui et al. [99] studied the properties of biological activated carbon (BAC) mass on COD elimination in LFL treatment. The COD reduction efficacy for reactors with 0, 100, and 300 g BAC dose per liter was 12.9, 19.6, and 27.7%, respectively [97]. Fazzino et al. [60] successfully removed bio-refractory substances (e.g., metals) from leachate by filtering active materials (i.e., zero-valent iron and granular activated carbon and Lapillus combinations). The pretreated leachate is then employed as nutrient solutions for market wastes in the sequential anaerobic digestion process to optimize the C: N and obtain a constant methane output of around 0.260 NL/gVS. Hedayati and Li [100] investigated the sorption capability of clinoptilolite-altered cationic surfactants-cetylpyridinium chloride-MC, didodecyldimethylammonium bromide, and hexadecyltrimethylammonium bromide-MC. These surfactants demonstrated improved hydrophobicity and enhancement in removing various polyaromatic hydrocarbons, with removal rates of >90%. In the Luo et al. [101] study, phosphoric acid-activated biochars made from rice husk biomass were effectively employed for leachate treatment through adoption, with high removal of color, contaminants, chemical oxygen demand, and NH_4_^+^-N. The mechanism of adsorption removal of pollutants from leachate is presented in Figure 6.

### 5.6. Alkaline Pretreatment-AD

Alkali pretreatment is one of the leading approaches for improving the biodegradation of complex materials is alkali pretreatment, which provides the most substantial advantages. The alkali pretreatment increases the effectiveness of the anaerobic digestion of complicated waste and receives special attention. In this case, NaOH is a preferred chemical and efficient at comparatively low dose levels in solubilizing munitions-grade nitrocellulose into soluble organic carbon forms [102]. Furthermore, NaOH, KHCO_3,_ and MgO can pretreat leachate. It has been proven that the alkali pretreatment of leachate may be used to address its acidic properties, which may hinder methanogenic biomass activity [16].

The first processes during alkali pretreatment are solvation and saponification, which cause solids to swell [103]. As a result, the specific surface area increases, and anaerobic bacteria may easily access the substrates. The COD solubilization is then boosted by simultaneous processes such as saponifying uronic acids and acetyl esters and neutralizing different acids generated by particle degradation [104]. According to Siciliano et al. [18], alkali pretreatment is used to balance the acidity of the leachate by adding basic chemicals. The goal was to reduce the volatile fatty acids/alkalinity ratio (VFA/ALK) to roughly 0.3, while retaining the pH between 6.5 and 8. When alkali techniques are used to pretreat substrates, it is crucial to remember that the biomass consumes some of the alkalies. Therefore, more significant alkali reagents may be necessary to achieve the desired anaerobic digestion increase [105]. Siciliano et al. [18] employed an alkali pretreatment of leachate supplied to the digester during the completed initial phase. The COD elimination was around 80% for applied loads equal to 24.5 kgCOD/m^3^d, and the highest daily biogas generation was approximately 9.3 L biogas/d (L reactor d). The CH_4_ proportion ranged between 70% and 78%, with a methane generation yield of 0.34–0.38 LCH_4_/gCOD removed. However, without alkali treatment, biogas output deteriorated due to VFA formation, and the pH was >6.5. The highest VFA- ALK ratio tolerated in the CSTR was 0.5 gCH_3_COOH/gCaCO_3_.

### 5.7. Photocatalysispretreatment-AD

Photocatalysis can be applied to pretreat landfill leachate in an environmentally responsible and cost-effective manner because it requires a brief contact period and is straightforward. There has been a lot of interest in photocatalytic degradation by semiconductors to increase the biodegradability and treatability of leachate. However, no complete study has been performed considering specifically anaerobic digestion of photocatalytic pretreated leachate, yet photocatalysis has been successfully utilized as the pretreatment of other substrates before anaerobic digestion. Because of its excellent efficacy, low production costs, and chemical inertness, TiO_2_ photocatalyst is one of many oxide semiconductors that is frequently employed in wastewater treatment [78]. TiO_2_ effectively eliminates bacteria and hazardous organic compounds from air and water, whereas Ag has been shown to have disinfecting properties [106]. Because of its biocompatibility, low toxicity, and inertness, TiO_2_ is often referred to as “the environmental white knight”, as reported in several studies [80,81]. These techniques are specific, so they do not necessitate any or very little precipitation, chemical additives, or biological activities. The approaches generate a variety of oxidants to eliminate aqueous contaminants.

Cai et al. [107] used cetyltrimethylammonium bromide bentonite–titanium dioxide photocatalytic system to pretreat the leachate, which improved the degradation of NH_3_–N and COD and achieved the removal of up to 37% and 82% in one hour, respectively [108]. To degrade contaminants from leachate, Yasmin et al. [109] used a photocatalytic technique with a TiO_2_/Ag composite and a biological treatment with a *Candida tropicalis* strain under visible light irradiation. It is interesting to note that following the nanocomposite treatment, the degradation rates for COD, TOC, and NH_4_^+^-N reached under ideal circumstances were 70%, 71.2%, and 49.1% percent, respectively. Following a biological treatment procedure, 90% COD, 84.6% TOC, and 75% NH_4_^+^-N were eliminated. Furthermore, average clearance rates of 95%, 63.8%, 50%, 83%, and 95%, respectively, of heavy metals, mainly Pb, Zn, Fe, Cu, and Cd, were significantly decreased.

**Figure 7:**
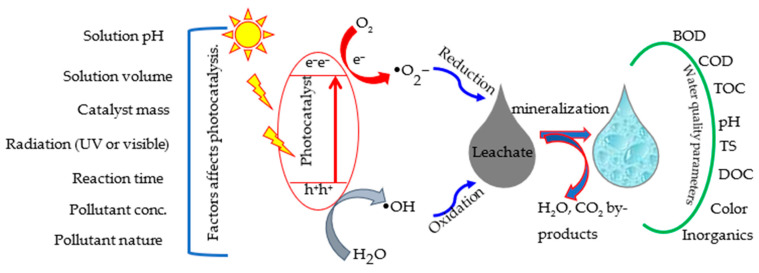
Mechanistic description, experimental and leachate properties affect the photocatalytic process.

The general mechanism of photocatalysis is outlined in Figure 7. The process begins with UV or visible light absorption, and electrons are promoted from the valence band to the conduction band to form electron-hole pairs (e/h^+^). Positive holes frequently oxidize organic molecules, causing them to degrade through oxidation. Furthermore, electrons primarily convert molecular oxygen to superoxide radical anions, resulting in various reactive oxygen species, including OH^o^, HO_2_^o^, and O_2_^−o^. Like other organic compounds, this reactive oxygen species could dissolve the refractory organic compounds in leachate and improve anaerobic digestion and biogas production [110].

### 5.8. Membrane Separation-AD

Anaerobic membrane bioreactors can be employed in the anaerobic process to enhance effluent water quality because membrane separation can remove pathogens and particles. Another benefit of membrane bioreactors is to minimize hydraulic retention time with extended solid retention, enhancing biogas production with a smaller footprint. This property of membrane bioreactor such that decoupling solid and hydraulic retention time can provide additional benefits, i.e., high COD removal and biogas production at psychrophilic temperatures up to 22 °C [51].

Membrane separation technology has been reported in various studies for leachate treatment. For instance, UASB-anoxic/oxic-ultrafiltration-nanofiltration, anoxic/oxic-membrane bioreactor-nanofiltration, and membrane bioreactor-nanofiltration/reverse osmosis [111]. Membrane bioreactors, which combine microfiltration or ultrafiltration membranes with biological reactors, have gained popularity in recent years and are regarded as advanced treatment processes due to their high effluent quality and flexibility [112]. Studies on treatment processes have shown that membrane bioreactors outperform traditional biological treatment systems across various loading circumstances, particularly when treating leachate from old landfills [113]. However, applying high-loading circumstances, extended hydraulic retention time and solids retention time, and high concentrations of pollutants can promote membrane fouling. Furthermore, high humic and fulvic acids in leachate have been demonstrated to accelerate membrane fouling [114,115].

The simplest and most generally used strategy for treating leachate membrane concentrates is recirculation into a membrane bioreactor, owing to its low cost [116]. However, the repeated cycling of leachate membrane concentrates can cause salt deposition in the membrane, which can be detrimental to biological functions [117]. Saleem et al. [115] studied dynamic membrane filtration behavior for stabilizing landfill leachate in a bench-scale pre-anoxic and aerobic submerged dynamic membrane bioreactor. The bioreactor showed a considerable organics removal of 50–60% and ammonia oxidation between 80 to 90. Saha et al. [51] developed an integrated leachate bed-anaerobic membrane bioreactor for effective stabilization and biogas recovery at room temperature. At a HRT of 13 and SRT of 75 days, a high COD removal of 86% was realized in the reactor system under optimum circumstances. Significant biogas recovery of about 850 kWh per ton obtained together with high quality of membrane permeates containing low COD concentration but advantageously high concentration of nutrients without any particles, which may be reused for landscaping or liquid fertilizer.

## 6. Techno-Economic Analysis of Anaerobic Digestion and Policy Requirement

The pretreatment of leachate is a relatively novel idea; its price approximation is still based on laboratory-scale research data. Table 4 summarizes various research indicating combining leachate treatment with other pretreatment or pre-processing procedures to reduce overall process costs. The entire capital expense of anaerobic reactors is significantly large, ranging from a few hundred thousand dollars to millions of dollars. Nevertheless, unlike a biogas digester plant, most other waste treatment technologies, which may likewise need a significantly high capital, may not produce revenue. According to several feasibility studies regarding anaerobic digesters conducted in North America, the payback period spans 5–16 years. Therefore, government financial aid for clean energy production might considerably shorten the payback period [118].

The investment expenses of an anaerobic digestion process comprise land value, which in this case is considered agricultural land rather than the land designated for industries. The average cost of industrial-designated land is ~6 times higher than that of agricultural land [119]. When land is viewed as an industrial component, the lower price of land overestimates economic performance. The project’s lifespan is questionable due to a lack of long-term expertise with digesters. Given its size and construction, a well-designed and maintained digester is projected to have a project life of twenty years [120].

Adopting this environmentally friendly approach relies on a legislative framework that establishes and offers a financially appealing incentive for operating AD plants. The technology may thrive with the proper regulations, but it is too unstable to provide significant incentives for investments in renewable energy technologies [121]. It is recommended that biogas plants be viable without a grant to explore new revenue streams, such as digestate and heat, or cost reductions by forming a contract with arable farmers to supply them with RO concentrate in exchange for less expensive energy crops. However, given the uncertainty of RO treatment requirements and heat and digestate values, we can conclude that high capital and operation investments limit the viability of AD of wastes until and how subsidies are granted [120]. Integrating the anaerobic digestion process with other sustainable treatment methods could save money and make the procedure more cost-effective.

However, for the landfill plant, it was required to investigate practical and cost-effective candidates to consider the leachate treatment process. According to Ye et al. [122], the long-term operation of leachate in an integrated treatment process is expensive because of high operational costs i.e., above 50 RMB, equivalent to 7.35 US dollars for treatment per m^3^ leachate. These operating costs are associated with large amounts of chemical doses, such as methanol, carbon source for denitrification, alkaline and acidic agents, etc. According to the current study’s assumptions, combining photocatalysis and anaerobic digestion dramatically lowered chemical and electric usage. Because photocatalysis requires no additional chemicals, the catalyst can be reused multiple times. Similarly, solar light can be used instead of expensive UV light to activate catalysts, reducing power use. Consequently, a significant cost reduction could be achieved for the overall process.

Although anaerobic digestion is an old process, it is still being successfully applied commercially to produce energy. The fact that the process is regularly disrupted indicates that technological and process-related skills have room for development. Despite these drawbacks, the prospect of carbon pricing and increased consumer awareness of environmentally sound and sustainable agricultural production practices will surely increase the benefits of ongoing research with this technique [118].

**Table 4:** Techno-economical evaluation of anaerobic digestion of leachate with various pretreated options.

Substrate /Leachate Type	Treatment Technology	Economic Evaluation (Cost of Process per $/m^3^) *	Pretreatment Applied **	Economic Viability ***	Comments	Reference
LFL	MBR	9.33 $/m^−3^	−	−	Considering the commercial value of NH_3_, the cost of the AS/AB pretreatment technique was minimized by 47%. Furthermore, the energy cost for leachate heating can be reduced by using landfill biogas and solar energy.	[123]
	Air-stripping/absorption (AS/AB) + MBR	4.92 $/m^−3^	+	++		
Composting Leachat	Struvite crystallization	8.84 $/m^−3^	−	−	Compared to struvite crystallization using pure chemicals without cycling, the economic analysis revealed that SPCT could save 59.0% of the processing cost.	[124]
	Thermal hydrolysis + Struvite pyrolysate cycling technology (SPCT)	4.24 $/m^−3^	+	++		
Municipal solid waste leachate	MBR	10.55 $ /m^−3^	−	−	Energy usage and external carbon sources account for most MBR system operation costs.	[125]
	steam-stripping pretreatment + MBR	1.86 $/m^−3^	+	++		
LFL	Mechanical vapor recompression (MVR)	6.93 $/m^−3^	−	+	The cost of treatment was significantly decreased by coagulation pretreatment.	[122]
	Coagulation + Mechanical vapor recompression	5.03 $/m^−3^	+	++		

* The cost of the process has been unified as price in dollars, adopting the current rate in the international market; ** Pretreatment Applied: (+Yes) (−No); *** Economic viability: (++High) (+Medium) (−Low).

## 7. Conclusions and Future Perspectives

The landfill leachates of dumpsite waste are a complex mixture of inorganic and organic substances, and the available treatment solutions are ineffective and expensive, posing a severe environmental danger. Leachate is usually produced in landfill, and its discharge after treatment will continue as a severe issue that must be addressed. Various landfill sites worldwide are either in operation or have previously closed and will continue to produce leachate for many years. As a result, the optimal technique or combination of different methods for treating leachate is required to be selected, preferably one that meets the criteria of effectiveness, low cost, ease of operation, and durability. A diverse range of alternative technologies was investigated in the literature summarized in the current review. Given the benefits of these methods, they can be used as pretreatment of leachate before the anaerobic digestion process. The following are some of the essential aspects of pretreatment:

(1) The hydrolysis of LFL in an anaerobic reactor system was significantly assisted by optimal pretreatment before anaerobic operations.
(2) The pretreatment of leachates enhanced the sCOD/tCOD ratio by converting insoluble organic matter to soluble COD.
(3) An anaerobic test fed with pretreatment LFL can increase biogas output and biogas or methane yield.
(4) Among various pretreatment methods, it has been observed that electrochemical and photocatalysis are the most feasible and adaptive procedures due to their high capability to degrade toxic organic and inorganic pollutants. Moreover, these methods are highly cost-effective because the material can be reused and is highly stable.

The integration of pretreatment with anaerobic processes for leachate treatment can improve performance and yield more biogas with greater methane concentration. The approach might potentially be used to improve the anaerobic treatability of other complicated industrial wastewater, particularly those with high organic content. In a future perspective, the appropriate use of pretreatment and optimization is required, that in other case pose difficulties in predicting and identifying safety and environmental risks. Thus, future research should consider the potential risks of the pretreatment method. Clean energy generation will be more efficient if using integrated technologies, i.e., pretreatment-AD, where the leachate may provide a significant share in energy recovery.

## Figures and Tables

**Table 2:** Anaerobic digestibility of leachate, degradation potential, and methane yield.

Leachate Type	Leachate Age *	Anaerobic Reactor	Reactor Conditions	Degradation Efficiency (Organic Matter% Removal)	Methane/Biogas Yield (m^3^/L)	Ref.
Municipal Solid Waste leachate	Fresh leachate	Up-flow Anaerobic Sludge Blanket (UASB)	OLR:18.2 kgCOD/m^3^/d HRT: 50 d	89	0.0048	[41]
LFL	Fresh leachate	Batch Reactor (BR)	Temp: 37 °C HRT: 24 d	53	0.292	[42]
Pressed off leachate	NR	BR	OLD: 27.7 kg COD/m^3^/d Temp: 37 °C	60	7.1	[43]
Landfill leachate	Mature leachate	Anaerobic Submerged Membrane Bioreactor	OLR: 2.5 kg COD/m^3^/d HRT: 2 d	90	NR **	[44]
Leachate with food waste	Fresh Leachate	UASB	OLR: 15.8 g COD/l/d.	96	0.055	[45]
Raw Leachate With food waste	Middle aged leachate	BR	OLR: 41.8 g VS/L. HRT 35 d	NR	0.466	[46]
Food waste leachate	NR	Bench-scale Two-Stage System (BSTSS)	Temp: 37 °C HRT: 99 d	94.3	0.393	[47]
Municipal Solid Waste leachate + Sludge	Fresh leachate	Stirring Batch Reactor (SBR)	Temp: 55 °C HRT: 90 d pH: 6.4–7.4	81.8	0.1173	[48]
LFL	Fresh leachate	Two-stage Anaerobic Sequential System	HRT: 21 d pH: 5–11	81	0.320	[49]
LFL	Fresh leachate	Anaerobic bioreactor (fixed-structured bed)	Temp: 30 °C HRT: 24 d OLR: 7.1 gCOD/L/d	NR	0.30	[50]
	NR	leachate bed reactor—anaerobic membrane bioreactor system (LBR-AnMBR)	Temp: 21–22 °C Leachate recirculation rate: 4.4–13.2 L/h	86	0.31	[51]
LFL + Activated sludge	Middle aged	Continuous stirred tank reactors	Temp: 37 °C, OLR: 1.3 g VS/L/day Operation: 180 d	91.3	0.105	[52]
Food waste + Mature leachate	Mature leachate	Expanded granular sludge blanket reactor	Temp: 35 °C OLR: 23.6 g COD/L/d	80	5.87 (L/L/d)	[53]
MSW leachate	NR	Anaerobic biofilm digester (ABD)	OLR: 1.27 g COD/ L/d HRT: 30 d	95	17 (m^3^/d)	[54]
Landfill leachate	NR	anaerobic membrane bioreactor	Temp: 35 °C Operation: 185 d Working vol: 6.3 l	78	436 (ml/L approx.)	[55]
OFMSW + Leachate	Fresh leachate	Solid-state anaerobic garage-type digesters	HRT: 72 d OLR: 0.5 kg VS/m/d	56%	0.06 m3 CH4.kg VS	[56]
+ Landfill leachate	Mature	Semi-continuous anaerobic reactors	OLR: 1.0 gVS/L/d HRT: 20 d	NR	0.260 NL/ gVS added.	[57]
LFL + Synthetic wastewater	Mature	UASB	Temp: 37 °C HRT: 1 d OLR: 2 gCOD/L/day^−1^	93	0.035 L g-COD^−1^	[58]

* Leachate age is determined based on the value biodegradability index or reported information; ** NR: not reported.

**Table 3:** Removal of pollutants from leachate during various treatment processes.

Substrate	Treatment Process/System	Target Pollutant	Pollutant Removal Efficiency	References
Leachate membrane retentate	Electrochemical oxidation	TOC NH_3_–N Total nitrogen	70.1% 98.7%, 69.7%	[70]
OFMSW leachate	Anaerobic membrane bioreactors	Ca, Mg P	95%, 74%, 84%	[71]
Mixed landfill leachate	Full scale leachate treatment plant (biological + UF-NF-RO membrane)	Antibiotic resistance genes (ARGs)	ARGs reduced 3 to 6 orders	[72]
MSW leachate	Biological and photochemical processes	NH_3_–N Total nitrogen COD	81% 92% 72%,	[15]
Landfill leachate	Photocatalysis	1,4-dioxane	81%	[73]
Membrane concentrated landfill leachate	Photocatalysis	Humic acid Fulvic acid Benzene derivatives	71.83% (TOC basis)	[74]
Landfill leachate	Submerged anaerobic biofilm reactor	Fe Pb Ni	90% 3%, 13%	[75]
Landfill leachate	Ultrafiltration- biological treatment	Cr Ni COD	61.6% 34.3% 79.8%	[76]
Landfill leachate	anaerobic sequencing batch reactor coupled with sequencing batch reactor (ASBR-SBR)	TN COD	95% 90%,	[62]
Leachate (anaerobic digestion effluent)	Ammonia stripping pretreatment-Anaerobic digestion	NH_4_^+^N PO_4−_^3^−P COD SS	78% 99.9% 82.1% 91%	[77]

## Data Availability

Data sharing is not applicable to this article as no new data were created or analyzed in this study.
